# Outcome of balloon kyphoplasty for the treatment of osteoporotic vertebral compression fracture in patients with rheumatoid arthritis

**DOI:** 10.1186/s12891-016-1215-4

**Published:** 2016-08-24

**Authors:** Jihoon Shim, Kwanghyun Lee, Hunchul Kim, Byungjik Kang, Haewon Jeong, Chang-Nam Kang

**Affiliations:** Department of Orthopaedic Surgery, Hanyang University College of Medicine, 222 Wangsimni-ro, Seongdong-gu, Seoul, 133-792 Korea

**Keywords:** Rheumatoid osteoarthritis, Osteoporotic vertebral compression fracture, Kyphoplasty

## Abstract

**Background:**

Osteoporosis and osteoporotic fractures are widely known as complications of rheumatoid arthritis. Kyphoplasty (KP) is known as an effective treatment modality for reducing pain and correcting kyphotic deformity in osteoporotic vertebral compression fracture (OVCF). However, cutcomes of KP in rheumatoid patients are not well known. The purpose of the study was to investigate the clinical and radiological outcomes of balloon KP on OVCF in patients with rheumatoid arthritis.

**Methods:**

A total of 23 patients (31 vertebral bodies) with rheumatoid arthritis who received KP for OVCF and could be followed up for at least 1 year were examined. For clinical outcomes, visual analogue scale (VAS) and the Korean version of the Oswestry disability index (KODI) were evaluated. For radiological outcomes, changes in anterior vertebral height and local kyphotic angle were measured, alongside cement leakage, adjacent fracture, and the recollapse of cemented vertebra.

**Results:**

The anterior vertebral height was significantly restored after surgery compared with prior to surgery (*p* < 0.001). Cement leakage was found in 14 cases (45.1 %), and disc space leakage was prevalent (50 %), while vascular cement leakage was found in one case. Adjacent fracture was found in 3 patients (11.5 %). VAS for lumbago showed a significant decrease (*p* < 0.001) after surgery (VAS = 2.4) compared with that before (VAS = 8.1); it was somewhat increased after the 1-year follow-up (VAS = 2.8; *p* = 0.223). KODI also decreased (48.8 %) after surgery compared with before (84.6 %). However, it increased somewhat (49.9 %) after the 1-year follow-up.

**Conclusion:**

KP on rheumatoid arthritis patients for OVCF was effective for reducing pain in the early stage and restoring vertebral body height. Recollapse of the treated vertebral body was found relatively frequently alongside the correction loss of local kyphotic angle.

## Background

Rheumatoid arthritis (RA) is known as one of the most common inflammatory arthritis conditions, and it manifests in 0.5–1 % of the entire population [1]. Osteoporosis and osteoporotic fractures are also widely known as complications of RA [1, 2]. Patients with RA are twice as likely to develop osteoporosis compared to those without RA, which doubles or triples their risk of developing osteoporotic fracture and increases their risk 6.2 times for osteoporotic vertebral compression fractures (OVCF) [3, 4]. Several studies report that the risk of death of RA patients increases by 32 % when OVCF occurs [5], and it is important to decrease critical complications through appropriate treatment during the early stages. A recent systematic review on OVCF treatments [6], which compared kyphoplasty (KP), percutaneous vertebroplasty (VP), and conservative treatment, reports that in comparison with conservative treatment, KP was better in terms of pain reduction and kyphotic deformity correction and involves a lower rate of subsequent fractures. However, no studies have been done to address the outcomes of KP performed on OVCF patients with RA . We reported that height restoration from vertebral collapse was higher in RA patients treated with balloon KP; however, there have been no reported studies on the clinical results of balloon KP [7].

This study is an extension of one of our our previous studies and its purpose was to investigate the clinical and radiological outcomes of KP performed on RA patients with OVCF and describe the their condition in the 1-year follow-up after the KP procedure.

## Methods

**Fig. 1:**
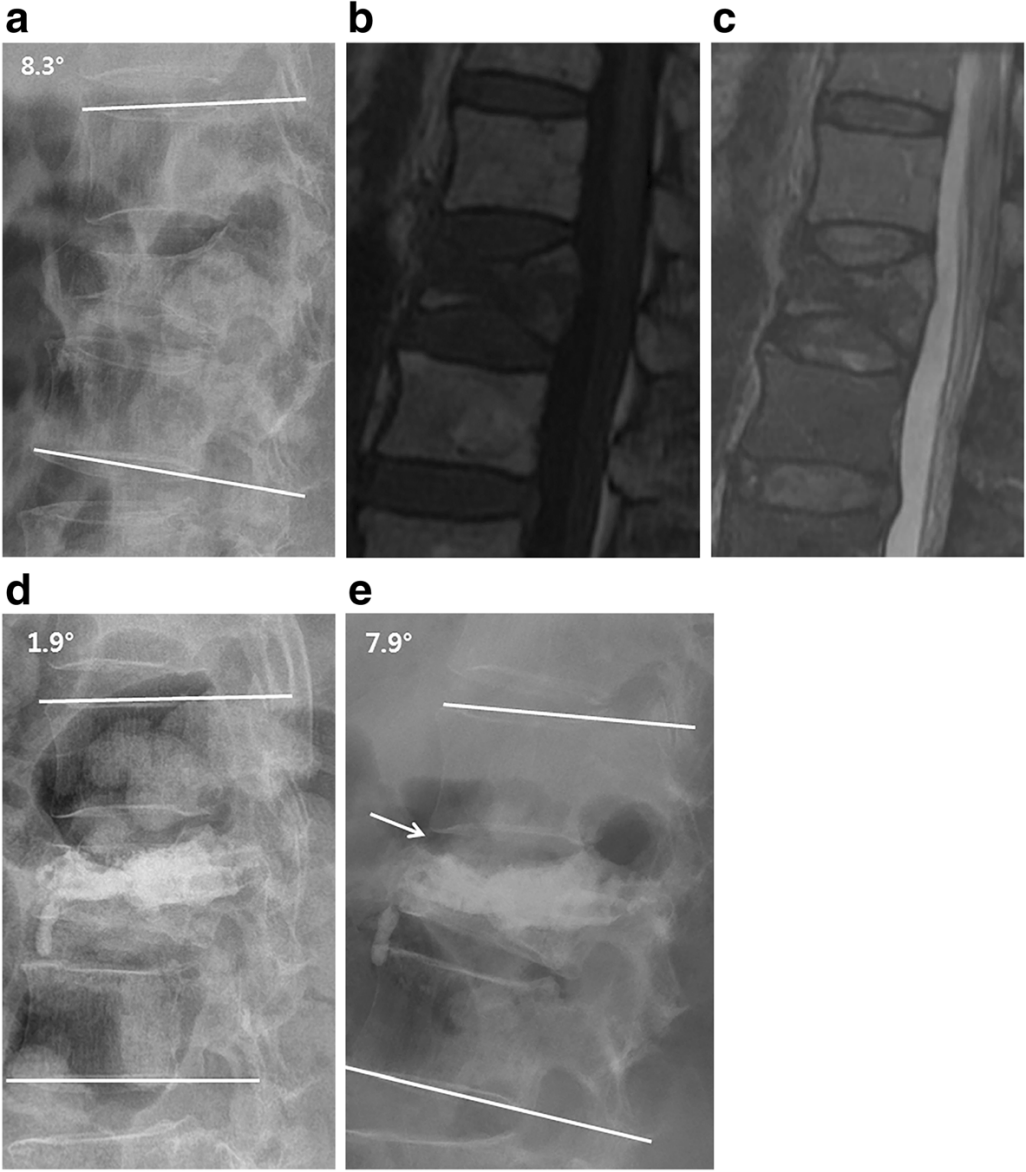
A 70-year-old female patient visited for the lumbago she experienced after she slipped at home. There was a suspected L1 vertebral body fracture based on the lateral plain radiograph (**a**). A recent fracture in T1WI and T2WI (**b**, **c**) was confirmed based on the MRI. After KP, the vertebral height was restored up to 67 % from 45 % before the procedure and the local kyphotic angle was improved from 8.3 ° down to 1.9 ° (**d**). However, at 1-year follow-up (**e**), osteolysis (*arrow*) was found around the cement inserted. The vertebral height reduced by 54 % and the local kyphotic angle worsened up to 7.9 ° compared with post-operational figures

This study selected and examined the following subjects: patients who were diagnosed with RA based on the American College of Rheumatology Standards at Hanyang University Hospital; patients who underwent KP for OVCF treatment from August 2007 to May 2013; and those we could follow up with for at least 1 year after the KP. The study was conducted after receiving an approval from the institutional review board approval (HYUH-2014-10-037). For an OVCF diagnosis, patients experiencing lower back pain due to a low impact injury and tenderness on the corresponding area and who presented with compression on the anterior vertebral body on a plain radiograph were examined by magnetic resonance image (MRI) and diagnosed as having a recent fracture (Fig. 1). Patients with a suspected fracture due to a high impact injury or a suspected pathological fracture due to an infection or tumor were excluded. Patients who complained about pain in everyday life, even after receiving conservative treatments such as stabilization in bed, drug treatment, and brace treatment for at least 3 weeks, and who manifested 30 % of the vertebral body compression rate in plain radiographs, were examined. A total of 23 patients and 31 vertebral bodies were included, and age at procedure, sex, body mass index (BMI), bone mineral density (BMD), time taken until procedure, and bone cement injection period and amount were checked using an electronic medical record (EMR). Bone density was measured using dual energy X-ray absorptiometry (DXA). Additionally, erythrocyte sedimentation rates (ESR) and c-reactive protein (CRP) levels were checked via blood tests before the procedure.

The procedure was performed by one surgeon (CNK) at the same medical institute under local anesthesia plus conscious sedation using the conventional bilateral transpedicular approach in the prone position. Prophylactic antibiotics were intravenously administered 30 min prior to the procedure and the patient’s blood pressure, pulse, and oxygen saturation were monitored. During bone cement injection, a C-arm fluoroscope was used to check against leakage, and the injector was drawn out once the injection was completed. The cement volume was calculated by adding the measured volumes of cement injected on both sides. Cement time was measured from the beginning of bone cement mixing until right before the injection. Walking was allowed immediately after the procedure and daily activities were permitted. Drug treatment for osteoporosis was also administered.

For clinical outcomes, a visual analogue scale (VAS) for lower back pain was used before and after the procedure, when discharged from hospital, and during the 1-year follow-up. The Korean version of the Oswestry disability index (K-ODI) [8] was used to assess disability. In addition, cardiopulmonary complications, neurologic complications, and infections related to the procedure were investigated.

The radiological measurements were taken twice at intervals of two weeks by a spine fellow (JHS) and an orthopaedic resident (BJK) both of whom had received training for the procedure plan previously but did not participate in the procedure. The PACS π View Star® (Infinitt, Seoul, Korea), a digital measuring program, was used to examine the compression rate of the fractured vertebral body and the trend of change in local kyphotic angle before and after the procedure and during the 1-year follow-up. Measurements were taken from the image of the vertebral bodies at maximum zoom, above and below the fractured vertebral body, in order to reduce the error between measurements.

For the compression rate on a vertebral body, the ratio of the anterior vertebral height to the average height was calculated using the lateral plain radiographs shot above and below the fracture in a standing or sitting position. Considering errors in measurement, when the vertebral body compression rate obtained from the 1-year follow-up is 5 % or more lower compared with that obtained after the procedure, the authors made a direct comparison between the image and the 1-year follow-up image on the same screen. Cases in which the vertebral body height had decreased and the bone absorption around the inserted bone cement (polymethyl methacrylate; PMMA) was noticeable were categorized as cases of recollapse (Fig. 1). For local kyphotic angle, the Cobb’s angle formed by the superior endplate of the vertebral body above the fractured vertebral body and the inferior endplate of the vertebral body below the fractured vertebral body was examined. As the thoracolumbar curve, which became the reference, varied depending on the location of the fracture, the difference between measurements taken before and after procedure and between those taken after procedure and during the 1-year follow-up were determined. For cement leakage, the positions were recorded separately as anterior, posterior, superior, inferior, and lateral, and if a posterior (spinal canal) leakage was suspected, a CT scan was performed to determine the degree of encroachment. By comparing this with the post-operational images radiologically, the adjacent fracture was examined using an MRI in cases of suspected recent adjacent fracture and clinically suspected cases where compression on the vertebral body was not noticable.

The SPSS v18.0 program was used for statistical analysis. A repeated ANOVA and then pair-wise comparison was used when comparing VAS, ODI, vertebral collapse rate, and local kyphotic angle obtained before and after the procedure and from the 1-year follow-up. A Mann-Whitney test was performed to compare the degree of bone density, cement injection, and local kyphotic angle between the recollapse group and the non-recollapse group. The intraobserver and interobserver reliability were verified with Cronbach’s alpha coefficient.

## Results

Patients in the study included 4 men and 19 women aged 70.7 ± 6.6 (54–82) on average (Table 1). Initial fractures included 13 cases of L1 (42 %), 6 cases of L2 (19 %), 3 cases of T12 (10 %), 2 cases of T11 (6 %), 4 cases of L4, 2 cases of L3, and 1 case of T8, which mostly appeared on the thoracolumbar junction. In addition, 2 simultaneous OVCFs were found in 4 cases and 3 simultaneous OVCFs were observed in one case. In most cases, the procedure was decided upon after conservative treatment had been given for 3 weeks after the injury; in some cases, the symptoms worsened with conservative treatment and the procedure was performed many months later. The time taken from the initial injury to procedure was 6.7 ± 3.6 weeks and the average follow-up period was 18.2 ± 4.3 months. Bone cement (PMMA) injection volume was 6.4 ± 1.8 cc per vertebral body and the injection time was 10.5 ± 1.4 min.

**Table 1:** Demographic data and clinical characteristics

Baseline presentation	23 patients (31 cases)
Age	70.7 ± 6.6
Gender (M:F)	4:19
Fractured level (N/%)	
T8	1(3 %)
T11	2(6 %)
T12	3(10 %)
L1	13(42 %)
L2	6(19 %)
L3	2(6 %)
L4	4(13 %)
BMI (kg/m^2^)	24.4 ± 3.6
Duration (week)	6.4 ± 3.6
BMD (g/cm^2^)	0.55 ± 0.1
Cement volume (ml)	6.4 ± 1.8
Cement time (min)	10.5 ± 1.4

Values are given as mean ± SD. T indicates thoracic spine; *L* lumbar spine, *BMI* body mass index, *BMD* bone mineral density

The intraobserver reliability for the vertebral body compression rate and local kyphotic angle, represented by the Kapa Coefficient, were 0.84 (95 % CI, 0.71–0.97) and 0.88 (95 % CI, 0.77–0.99) respectively, which were outstanding results; interobserver reliability, which is also measured using the Kapa Coefficient, were 0.75 (95 % CI, 0.60–90) and 0.76 (95 % CI, 0.66–0.86) respectively, which were also exceptional.

Vertebral height before procedure was 56.3 ± 13.0 % and there was significant height restoration (*p* < 0.05) of up to 74.5 ± 12.4 % after KP (an average increase of 18.1 %). Also, vertebral height was significantly higher in the 1-year folow-up than before the procedure (*p* < 0.05). However, it declined by 4.3 % on average to 70.1 ± 12.8 % according to the 1-year follow-up (*p* < 0.05; Table 2). Among the 10 cases where the measurement taken before procedure decreased more than 5 % in the measurement obtained during the 1-year follow-up, osteolysis was noticeably found around PMMA in 6 cases (19.4 %) and was categorized as recollapses (Fig. 1). When comparing the recollapse group and the non-recollapse group, there were no changes in demographic data, BMD, erythrocyte sedimentation rate (ESR), and c-reactive protein (CRP) level, and there were no significant differences in cement volume, cement time, and cement leakage. There were significant differences between the two groups in terms of the decrease in vertebral height calculated from the difference between the height measured after the procedure and the heigh from the 1-year follow-up. Furthermore, the correction loss of local kyphotic angle revealed significant differences between the two groups. On the other hand, there were no significant differences between two groups in regards to clinical outcomes (Table 3).

**Table 2:** Clinical and radiological outcomes

	Preoperation	Postoperation	1 year follow up	*P* value (Preop.-Postop./Postop.-1 year FU)
Radiologic outcomes				
Vertebral height (%)	56.3 ± 13.0	74.5 ± 12.4	70.1 ± 12.8	<0.001/<0.001
Differences of local kyphotic angle(°)		3.9 ± 4.3	4.0 ± 3.7	<0.001/<0.001
Clinical outcomes				
VAS	8.1 ± 0.7	2.4 ± 0.8	2.8 ± 2.1	<0.001/0.223
ODI	38.1 ± 3.9	22.0 ± 4.8	22.4 ± 7.6	<0.001/0.796

Values are given as mean ± SD. VAS indicates visual analog scale; *ODI* Oswestry Disability Index

**Table 3:** Comparative results between recollapse and non-recollapse group

	Recollapse group	Non-recollapse group	*P*-value
Age(years)	72.5 ± 4.3	69.3 ± 7.1	0.391
Sex			0.901
Female (n)	5	15	
Male (n)	1	2	
BMI (kg/m2)	23.0 ± 3.9	25.1 ± 3.3	0.127
BMD (g/cm2)	0.53 ± 0.13	0.56 ± 0.08	0.274
ESR (mm/h)	35.0 ± 17.0	35.4 ± 25.8	0.695
CRP (mg/dL)	1.17 ± 0.62	1.96 ± 2.49	0.764
Cement volume (ml)	6.2 ± 2.1	6..5 ± 1.8	0.835
Cement time (min)	10.9 ± 1.4	10.4 ± 1.4	0.473
Cement leakage (n)	9/24	5/7	0.112
Loss of vertebral height (%)	9.3 ± 4.9	2.8 ± 3.4	0.001
Loss of kyphotic angle (°)	7.6 ± 3.5	3.3 ± 3.2	0.008
VAS (point)	−0.8 ± 1.4	−0.3 ± 1.9	0.595
ODI (point)	−0.35 ± 8.27	3.0 ± 12.6	0.502

Values are given as mean ± SD. *BMI* indicates body mass index, *BMD* bone mineral density, *ESR* erythrocyte sedimentation rate, *CRP* c-reactive protein, *VAS* difference of visual analog scale between postoperation and 1 year follow up, *ODI* difference of Oswestry Disability index between postoperation and 1 year follow up

An adjacent fracture was found in 3 cases (9.6 %), all of which where located above the treated vertebral body. In 2 out of the 3 cases, KP was also performed on the corresponding segment, with the patient showing signs of improvement after conservative treatment in one case. Local kyphotic angle showed approximately 3.9 ° of deformity correction after procedure, compared with that prior to procedure (*p* < 0.05); however, there was also a 4.0 ° correction loss of kyphotic angle noted in the 1-year follow-up (*p* < 0.05; Table 2). There was no significant improvement observed in the 1-year follow-up in comparison with that before the procedure (*p* = 0.235).

Bone cement leakage appeared in 14 cases (45.1 %), with disc space leakage being the most prevalent. Leakage was found in seven cases, close to the vertebral body in 5 cases and spinal canal leakage in two cases. There were no cases of cardiopulmonary adverse event associated with cement leakage and no neurological symptoms were observed in two cases of spinal canal leakage.

In regard to clinical outcomes, pain in the early stage decreased significantly after KP (VAS = 2.4; *p* < 0.05) compared with that before KP (VAS = 8.1). Also, the VAS score obtained during the 1-year follow up was significantly lower than the one before KP (*p* < 0.05). There was no significant difference between after KP and during one-year follow up VAS scores (*p* = 0.223). ODI decreased significantly after procedure (48.8 %; *p* < 0.05) compared with prior to procedure (84.6 %). Also, after 1-year follow-up, there was significantly decreased than before procedure (*p* < 0.05). However, it increased somewhat up to 49.9 % after 1-year follow-up (*p* = 0.811). There were no medical complications during the procedure and no cases of infection in the operated areas.

## Discussion

It is known that bone loss is caused by the interaction between a number of factors in RA patients including activity of the disease, age, physical activity, duration of the disease, and blood test figures [9–11]. It was also reported that overall bone strength decreases due to various factors and the risk of osteoporotic spinal fracture increases 6.2-fold [3, 4]. Such a decrease in bone strength may lead to frequent cement leakage through a fractured vertebral body wall or endplate during kyphoplasty, and to an increase in the frequency of complications, such as an adjacent fracture or a recollapse in the treated vertebral body. However, there are no reported outcomes of KP in RA patients. As it is possible that additional surgeries may be required due to complications, which may cause worse clinical outcomes compared with conservative treatment, it is meaningful to analyze the efficacy and safety of KP on RA patients with OVCFs. It may also help in determining the direction of treatment.

Voggenreiter [12] reported that vertebral height restoration is possible through balloon inflation and dynamic fracture mobility in kyphoplasty, which is supported by a number of clinical studies [13–15]. Lovi et al. [14] compared KP and VP and reported that the vertebral height restoration effect of KP was relatively high in 2-year follow-ups. This study achieved an average 18.1 % of significant vertebral height restoration in the post-operative radiograph. However, it also revealed a 4 % loss of height restoration at the final follow-up. A recent biomechanical study [16, 17] that used a cadaver reported that the recollapse of the vertebral body was finally observed when 100,000 repetitive loads were applied after KP, explaining that a recollapse occurs when vertebral bodies above and below the PMMA are continuously compressed, or when the cancellous bones of the vertebral bodies above and below with relatively less strength are absorbed through the stress-shield effect, as the main load is transferred through the bone cement, which has greater strength, rather than the cancellous bone of the vertebral body. Kim et al. [18] reported a recollapse rate of 12.5 %, whereas it was 22.5 % in this study, a relatively higher value than the figures suggested in existing studies. Although there were no factors showing significant differences between the recollapse group and the non-recollapse group, RA patients had an overall decrease of bone strength due to multifactorial causes. In addition, they showed a decrease in bone quantity as well as bone quality, and it would be possible to suggest that the recollapse could have been amplified merely with the repetitive application of load in everyday life.

In our study, the recollapse group showed worse clinical outcomes than the non-recollapse group after procedure and at 1-year follow-up. Kim et al. [18] reported that clinical symptoms worsened when there was a recollapse of the vertebral body, which our study concurs with. But, there was no statistical significance between two groups. Since this was a short term follow-up outcome obtained from a small sample of patients, further study of medium to long term clinical outcomes using more subjects is required. Also, in our study, the correction loss of local kyphotic angle was significantly high in the recollapse group. It is thought that this progression of the deformity caused chronic symptoms in the medium to long term follow-ups and influenced clinical outcomes.

For sagittal balance correction, Predhan et al. [19] recently reported that the partial success of the kyphotic angle correction of a collapsed vertebral body would not influence the restoration of the multilevel sagittal angle and sagittal balance. In this study, the local kyphotic angle showed an improvement of 3.9 ° after surgery. However, it also showed a correction loss by 4 ° during the 1-year follow-up compared with that after kyphoplasty. Local kyphotic angle worsened compared with that before surgery in 3 patients (11.5 %), even though there was no recollapse or adjacent fracture. Kawaguchi et al. [20] reported in their study about RA that the facet joint of the spine is a typical target joint of RA. The role of this facet joint is to restrict movement during extension and axial rotation, which is considerably important. Adams et al. [21] reported that the capsular ligament acted as the strongest resistance when a certain flexion moment was applied. Hence, destruction of facet joint and capsular ligament in RA patients was considered as one of the causes that dynamically accelerated local kyphotic deformation.

During kyphoplasty, bone cement leakage is common (from 11.3 to 33 %) [22, 23]. There is a report that states that bone cement leakage increased three-fold when the end plate of the vertebral body was fractured, and the risk of adjacent fracture increased four-fold when the leakage took place within the disc [24]. In this study, the cement leakage took place in 14 cases (45.1 %), which was higher than that in previous studies; however, the amount leaked was quite small. Vascular cement leakage was found in only one case, and there were no serious adverse events in any case. In 2 cases (6.4 %), the leakage took place inside the spinal canal, but it did not cause any neurological symptoms. Authors injected cement by checking the distributional shape of the bone cement through the viewfinder when the cement was polymerized; it was left for at least 10 min (with an average of 10.7 min), where became quite viscous and could prevent excessive leakage during the fracture of the end plate. Thus, we suggest that even if there is a cement leakage, complications including pulmonary embolism were not related to cement leakage because of the high viscosity and more rigid nature of the cement. Furthermore, disc space leakage was found in 7 cases (22.5 %) and there was no case of adjacent fracture. There are still many disputes about whether disc space leakage increases the load transfer to adjacent vertebral bodies. Although not to be included in this study, it is proposed that sagittal balance, the leakage amount and distribution aspect of PMMA, and bone mineral density of the adjacent vertebral bodies affect load transfer at adjacent vertebral bodies [25].

Kayanja et al. [26] performed a biomechanical study on the transfer of load onto the adjacent segment after bone cement reinforcement. They reported that it did not increase the load transfer to an adjacent segment within the scope of a physiological load transfer. Recently, Papanastassiou et al. [6] reported that the rate was relatively low when KP was performed for overall subsequent fractures (11.7 %) compared with cases of non-surgical management (22.7 %). Both bone density and quality deteriorated in RA patients and a relatively high incidence rate of adjacent fractures was expected, compared with preceding studies. On the other hand, an adjacent fracture occurred in 3 cases (9.6 %), matching the results of preceding studies.

In a recent systemic review [6] which compared the effect of KP, VP, and conservative management for OVCF, KP, and VP demonstrated an improved pain reducing effect over conservative treatment and KP showed enhanced results for quality of life improvement over VP. Considering the RA patients examined in this study, the pain reduction in the early stages after KP showed a significant improvement as the VAS score decreased by 5.7 points after surgery (*p* < 0.001). Ledlie et al. [22] reported in the 2-year follow-up study performed after KP that the VAS score improved, going down from 8.9 points before surgery to 2.8 points after surgery, and again down to 1.5 after during the 2-year follow-up. However, Coumans et al. [27] also reported that the VAS score did not demonstrate a statistically significant difference between the score after KP and during the 1-year follow-up. Similarly, in this study, the VAS score decreased to 2.4 after KP. VAS scores did not have significant changes between after KP and during the one-year follow up (*p* = 0.223) Clinical improvement was noted after the operation, and it persisted through the 1-year follow-up despite a statistically insignificant increase. This is thought to be attributable to the inclusion of pain related to the correction loss observed radiologically, and was also a subjective reaction of chronic back pain [28] due to the lumbar spine involvement in RA symptoms. KODI improved down to 48.8 % after surgery compared with that before surgery (84.6 %), and down to 49.9 % in the 1-year follow-up compared with post-operational results. However, no statistically significant changes were observed.

This study had some limitations. First, it was a retrospective study, and the number of patients included was small. Second, the study included ESR and CRP only while the evaluation of subjective pain due to the fracture in relation to the activeness of RA was excluded; in addition, it did not evaluate bone quality. Third, it could not reflect global sagittal imbalance, as it was not possible to shoot the entire view of all patients. Finally, it could not quantitatively compare the dosage of various medications used for RA. We suggest that an additional well-designed prospective study including these issues is necessary in the future.

## Conclusion

Unlike the hypothesis of the authors, KP on RA patients with OVCF was effective for reducing pain in the early stages and restoring functional recovery. Although there were frequent leakages of bone cement, there were no complications related with these leakages. However, in terms of radiology, there were frequent findings of a recollapse of the treated vertebral body and a correction loss of local kyphotic angle at the 1-year follow-up. Therefore, KP on RA patients with OVCF requires close follow-ups in cases of recollapse. Also, it is necessary to explore the relationship between medium to long term radiological and clinical results in the future.

## Abbreviations

BMD: Bone mineral density
BMI: Body mass index
CRP: C-reactive protein
DXA: Dual energy X-ray absorptiometry
EMR: Electronic medical record
ESR: Erythrocyte sedimentation rates
K-ODI: Korean version of the Oswestry disability index
KP: Kyphoplasty
MRI: Magnetic resonance image
OVCF: Osteoporotic vertebral compression fracture
PMMA: Polymethyl methacrylate
RA: Rheumatoid arthritis
VAS: Visual analogue scale
